# Supplementary material for: IP3 receptor isoforms differently regulate ER-mitochondrial contacts and local calcium transfer
Source: Nat Commun. 2019 Aug 19;10:3726. doi: 10.1038/s41467-019-11646-3 (PMC6700175; doi:10.1038/s41467-019-11646-3)
Supplement: Supplementary file 1 — Supplementary Information [file 41467_2019_11646_MOESM1_ESM.pdf]

IP_3 receptor isoforms differently regulate ER-mitochondrial contacts and local calcium transfer

Bartok et al.

Supplementary figures

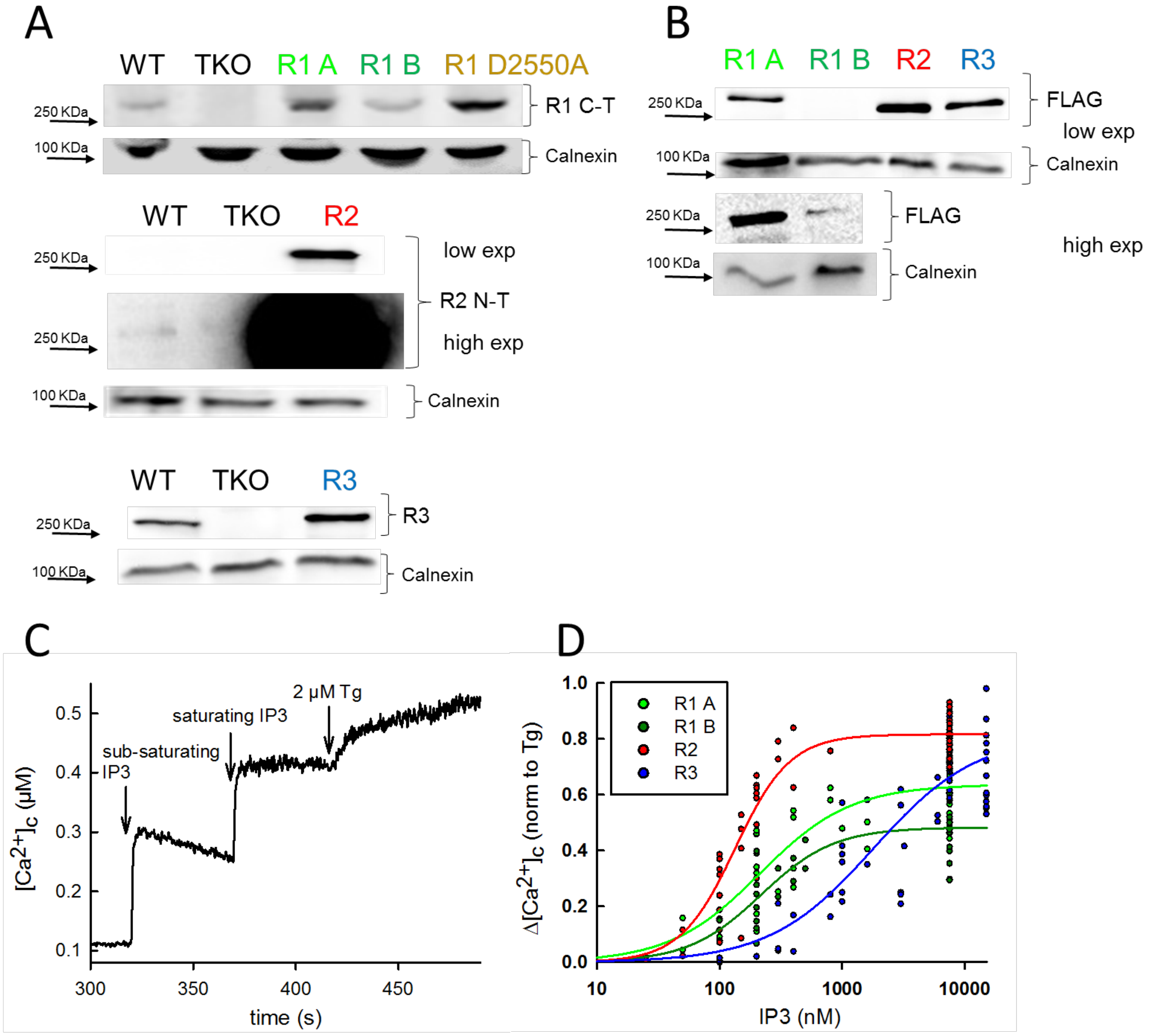

Supplementary Figure 1. Mammalian IP3R isoforms are expressed in comparable levels in DT40 cells

A, Representative immunoblotting of DT40 cell lysates with different IP3R expression. IP3R1 (top) IP3R2 (middle) and IP3R3 (bottom) proteins were detected with respective isoform specific antibodies. Expression levels in the rescue clones appeared similar (R1B) or higher (R1A, R2, R3) than the endogenous IP3R isoforms in WT. However, it is unknown whether the anti-IP3R antibodies recognize with similar affinity the endogenous avian IP3Rs of the DT40 cells and the mouse and human IP3R used for the rescue. **B,** Representative immunoblotting of DT40 cell lysates rescued with FLAG-tagged mammalian IP3R isoforms. Anti-FLAG antibody was used for detection. **C,** IP_3 sensitivity for each IP3R isoform was tested with cuvette-based fluorimeter in suspensions of permeabilized cells. ER Ca^{2+} release was stimulated by adding a sub-saturating

and then a saturating IP_3 pulse followed by thapsigargin (Tg). Released Ca^{2+} was measured with fura2 in the intracellular medium ($[Ca^{2+}]_{cyto}$). Representative trace is shown for R3 cells. **D,** IP_3 dose-response curves of the DT40 cells expressing a single isoform of IP3R. Points are normalized to the full store discharge by Tg (N = 74, 55, 60 points, respectively from 6 independent experiments). Source data are provided as a Source Data file.

A

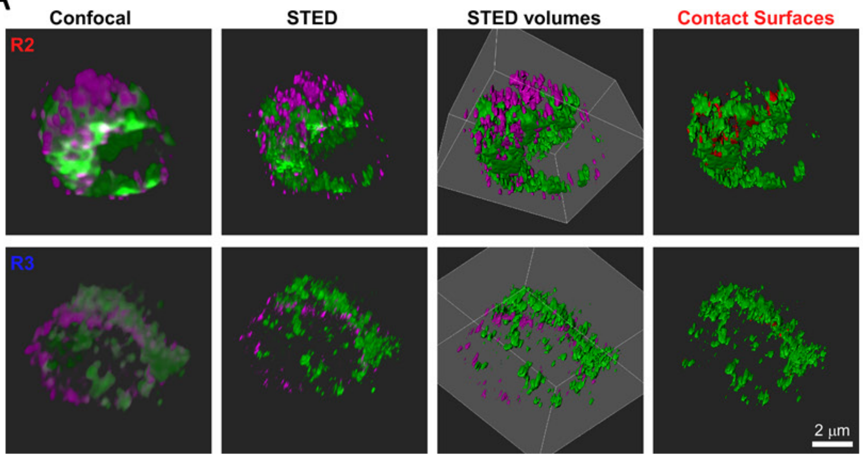

B

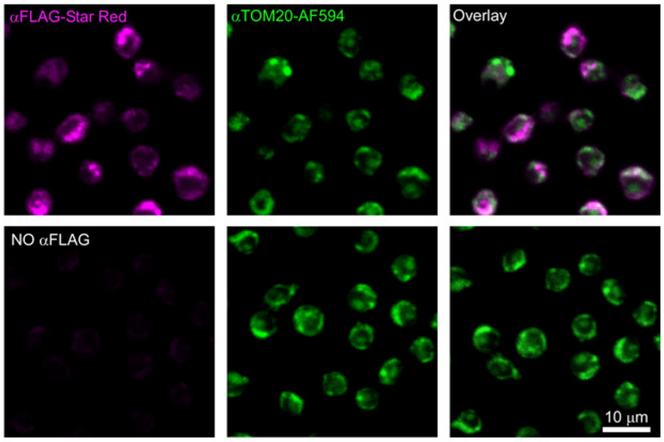

C

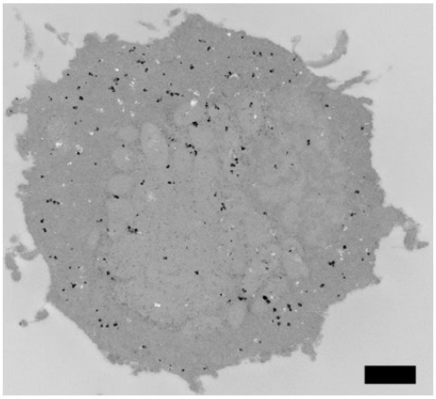

D

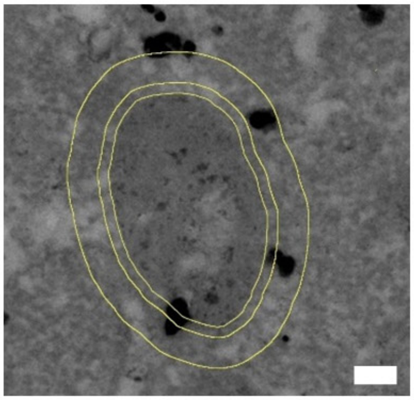

E

	TKO	S.E.M	R1 A	S.E.M	R2	S.E.M	R3	S.E.M
particle / μm^2 cyto	2.8	0.5	14.8	0.7	7.6	0.8	10.4	1.0
N gold in 100 nm halo norm to mito perimeter			13.27	1.76	11.86	2.12	7.87	0.74
N gold in 25 nm halo norm to mito perimeter			2.95	0.67	5.19	1.23	3.22	0.47
25 nm/100 nm (%)			20.34	3.29	35.14	6.06	40.37	4.92

Supplementary Figure 2. Accumulation of IP3 receptors at the ER-mito interface revealed by Immuno-EM and STED microscopy

A, IP3Rs (magenta) and OMM (green), anti-FLAG and anti-TOM20 antibodies, respectively, were imaged by STED microscopy. Places where the mitochondrial surface is in contact with IP3Rs are shown in red in the rightmost image. **B,** Confocal images of the IP3R2 sample used in (A) and the same cells stained without anti-FLAG primary antibody. **C&D,** Flag-tagged IP3Rs were labelled by anti-Flag nanogold particles followed by silver enhancement reaction. Images of whole cell sections **(C)** (8000X, scale bar = 1 μm) and mitochondria **(D)** (15000X, scale bar = 100 nm) were analyzed by ImageJ software. Nanogold particles were analyzed in the whole cell sections **(C)** and in the close environment (25 and 100 nm, yellow area selections) of the mitochondria **(D)**. **E,** Quantification of IP3R accumulation in the close vicinity of the mitochondria (0-25 nm). N = 6 TKO, 8 R1 A, 5 R2 and 6 R3 cells and 23 R1 A, 27 R2 and 52 R3 mitochondria in one experiment. Source data are provided as a Source Data file.

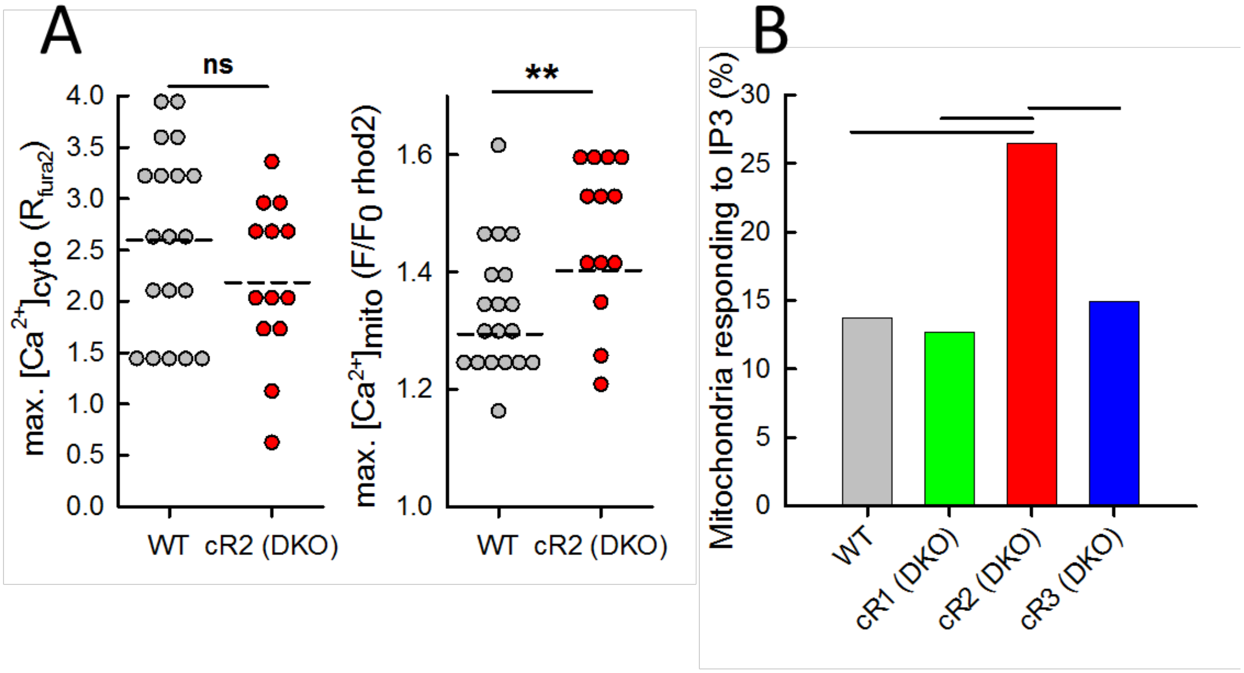

Supplementary Figure 3. Endogenous IP3R2 is the most efficient in delivering Ca^{2+} to the mitochondria

A, Maximal $[Ca^{2+}]_{cyto}$, (left) and maximal $[Ca^{2+}]_{mito}$ (right) signals **(**mean, dashed lines) in permeabilized WT and cR2 DKO cells (N = 19 and 13 runs, respectively). ** $p<0.01$ unpaired Student's T-test. **B,** Permealized DKO cells showing rapid mitochondrial Ca^{2+} uptake followed by 7.5 µM IP3 stimulus. Black lines represent $p<0.0001$ with Chi-square test (N = 611 (WT), 734 (cR1), 374 (cR2) 515 (cR3) cells from 3 independent experiments). Source data are provided as a Source Data file.

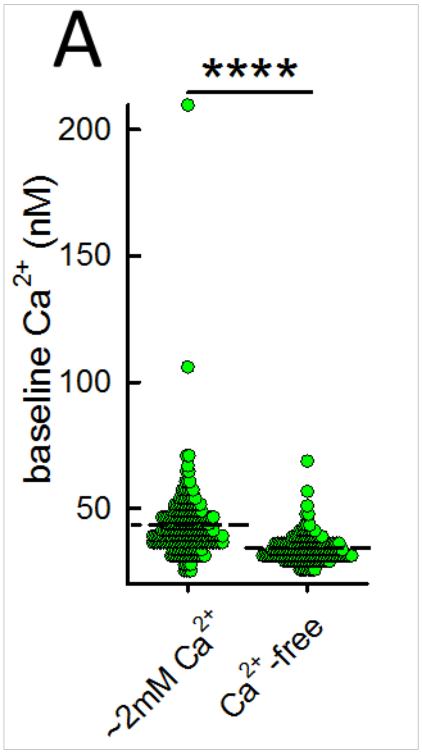

Supplementary Figure 4. Extracellular [Ca^{2+}] affects cytoplasmic baseline Ca^{2+} levels

A, [Ca^{2+}]$_{cyto}$ baseline levels measured with fura2 in the indicated extracellular [Ca^{2+}] solutions (mean, dashed line) in R1 A cells. **** $p<0.0001$, Student's T-test (N = 229 and 169 cells, respectively from 3 independent experiments). Source data are provided as a Source Data file.

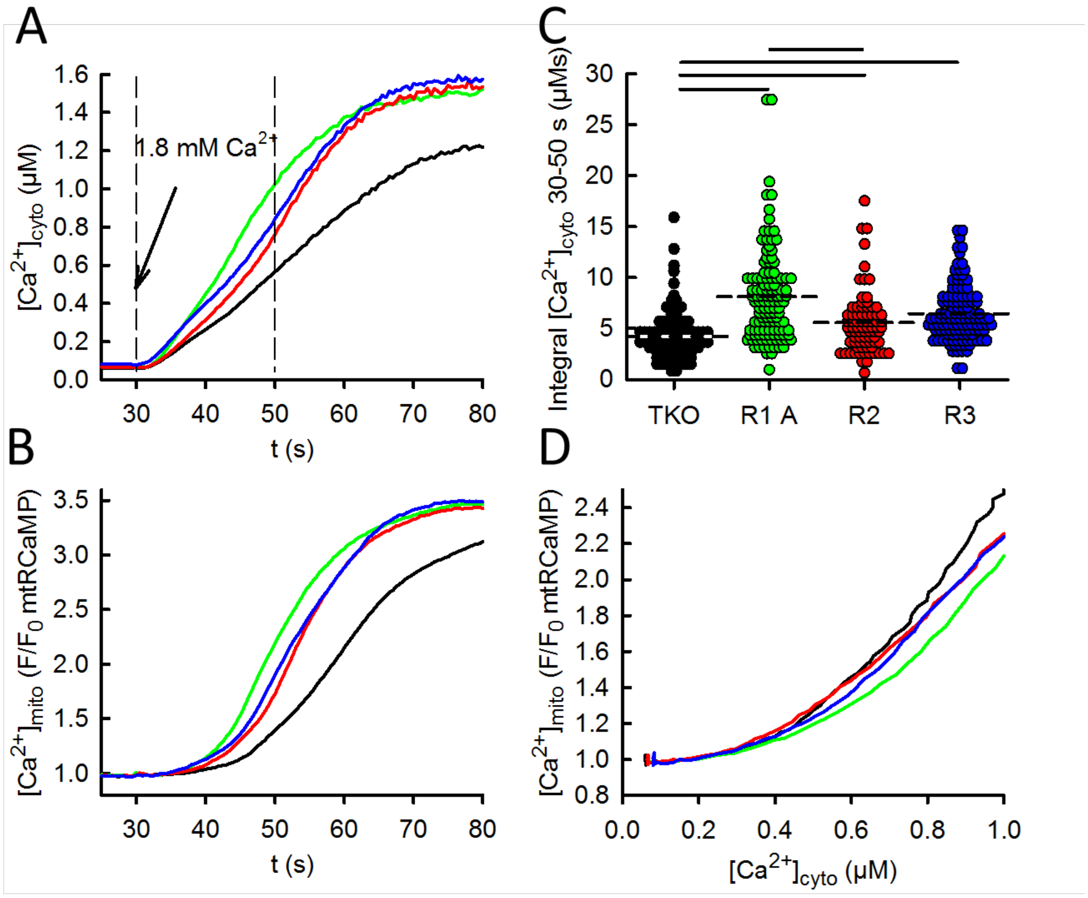

Supplementary Figure 5. IP3Rs facilitate store operated Ca^{2+} entry

Fluorescence wide-field imaging of $[Ca^{2+}]_{cyto}$ and $[Ca^{2+}]_{mito}$ in individual intact DT40 cells. **A,** Cytoplasmic $[Ca^{2+}]$ ($[Ca^{2+}]_{cyto}$) was measured with fura2 in Ca^{2+}-free extracellular solution. Cells were treated with 2 μM thapsigargin 10 minutes then with 500 nM trypsin 20 seconds prior the addition of 1.8 mM extracellular Ca^{2+}. Mean traces are shown (N = 98 (TKO), 111 (R1 A), 79 (R2), and 111 (R3) cells from 3 independent experiments) **B,** $[Ca^{2+}]_{mito}$ was measured with mtRCaMP simultaneously with $[Ca^{2+}]_{cyto}$. **C,** Initial phase of the cytoplasmic Ca^{2+} signal (mean, dashed line) was compared with one-way ANOVA with post-hoc Dunn method. Black lines represent $p<0.05$. **D,** Mitochondrial Ca^{2+} uptake in response to the cytoplasmic $[Ca^{2+}]$. Source data are provided as a Source Data file.
