# Supplementary material for: IP3 receptor isoforms differently regulate ER-mitochondrial contacts and local calcium transfer
Source: Nat Commun. 2019 Aug 19;10:3726. doi: 10.1038/s41467-019-11646-3 (PMC6700175; doi:10.1038/s41467-019-11646-3)
Supplement: Supplementary file 3 — Reporting Summary [file 41467_2019_11646_MOESM3_ESM.pdf]

nature research

Corresponding author(s): Gyorgy Hajnoczky

Last updated by author(s): Jun 5, 2019

Reporting Summary

Nature Research wishes to improve the reproducibility of the work that we publish. This form provides structure for consistency and transparency in reporting. For further information on Nature Research policies, see Authors & Referees and the Editorial Policy Checklist.

Statistics

For all statistical analyses, confirm that the following items are present in the figure legend, table legend, main text, or Methods section.

n/a	Confirmed	
☐	☒	The exact sample size (*n*) for each experimental group/condition, given as a discrete number and unit of measurement
☐	☒	A statement on whether measurements were taken from distinct samples or whether the same sample was measured repeatedly
☐	☒	The statistical test(s) used AND whether they are one- or two-sided<br>*Only common tests should be described solely by name; describe more complex techniques in the Methods section.*
☒	☐	A description of all covariates tested
☐	☒	A description of any assumptions or corrections, such as tests of normality and adjustment for multiple comparisons
☐	☒	A full description of the statistical parameters including central tendency (e.g. means) or other basic estimates (e.g. regression coefficient) AND variation (e.g. standard deviation) or associated estimates of uncertainty (e.g. confidence intervals)
☐	☒	For null hypothesis testing, the test statistic (e.g. *F*, *t*, *r*) with confidence intervals, effect sizes, degrees of freedom and *P* value noted<br>*Give P values as exact values whenever suitable.*
☒	☐	For Bayesian analysis, information on the choice of priors and Markov chain Monte Carlo settings
☒	☐	For hierarchical and complex designs, identification of the appropriate level for tests and full reporting of outcomes
☒	☐	Estimates of effect sizes (e.g. Cohen's *d*, Pearson's *r*), indicating how they were calculated

Our web collection on statistics for biologists contains articles on many of the points above.

Software and code

Policy information about availability of computer code

Data collection	PTI Felix GX Photon Technology Intl., Inc. Version 4.2; Zen Carl Zeiss Microscopy GmbH Version 2.3; Vutara SRX Bruker Versions 5.23, 6.00, 6.01; MetaMorph Molecular Devices Version 7.6; Spectralyzer In-house
Data analysis	Sigmaplot Systat Software, Inc. Version 12.5; ImageJ National Institutes of Health, USA Fiji (IJv1.52); Python Open source (Anaconda, Inc.) Version 2.7; ER Mito Analysis (ImageJ plugin) In-house (Open source) http://sites.imagej.net/MitoCare/; Excel Microsoft 2010 and 2016

For manuscripts utilizing custom algorithms or software that are central to the research but not yet described in published literature, software must be made available to editors/reviewers. We strongly encourage code deposition in a community repository (e.g. GitHub). See the Nature Research guidelines for submitting code & software for further information.

Data

Policy information about availability of data

All manuscripts must include a data availability statement. This statement should provide the following information, where applicable:

- Accession codes, unique identifiers, or web links for publicly available datasets
- A list of figures that have associated raw data
- A description of any restrictions on data availability

The datasets generated during and/or analyzed during the current study are available from the corresponding author on reasonable request.

Field-specific reporting

Please select the one below that is the best fit for your research. If you are not sure, read the appropriate sections before making your selection.

☒ Life sciences ☐ Behavioural & social sciences ☐ Ecological, evolutionary & environmental sciences

Life sciences study design

All studies must disclose on these points even when the disclosure is negative.

Sample size	We did not use any statistical method to pre-determine sample sizes. Our goal was to obtain a reasonable sample size during each experiments, depending on the nature of the experiment. in TEM and immunofluorescence imaging, a minimum of 10 cells were analyzed per condition per fixation. FRET and Ca2+ imaging was performed with a large field detector, therefore the number of individual cells were multiples of the performed runs. For these experiments 5-10 independent runs were performed per experimental day.
Data exclusions	No data points were excluded.
Replication	All main figure data was obtained in at least 3 independent sets of experiments.
Randomization	Measurements (FRET, cytosolic and mitochondrial Ca2+ levels,) performed on different cell lines on the same day were randomized to avoid the detection of possible differences caused by different incubation times (e.g. following transfection or cell plating).
Blinding	Electron micrograps were analyzed blinded. Other experiments were not performed blinded.

Reporting for specific materials, systems and methods

We require information from authors about some types of materials, experimental systems and methods used in many studies. Here, indicate whether each material, system or method listed is relevant to your study. If you are not sure if a list item applies to your research, read the appropriate section before selecting a response.

Materials & experimental systems

n/a	Involved in the study	
☐	☒	Antibodies
☐	☒	Eukaryotic cell lines
☒	☐	Palaeontology
☒	☐	Animals and other organisms
☒	☐	Human research participants
☒	☐	Clinical data

Methods

n/a	Involved in the study	
☒	☐	ChIP-seq
☒	☐	Flow cytometry
☒	☐	MRI-based neuroimaging

Antibodies

Antibodies used

FLAG Sigma F1804-200UG 088K6018
IP3R3 BD Transduction laboratories 610312 51627
IRDye 800CW Donkey Anti-Rabbit LiCor 926-32213 C60712-05
AF647 Rabbit Anti-Mouse Thermo Fisher A-21239 1774710
CF568 Goat Anti-Rabbit IgG Biotium 20103-1 16C0422
AF647 Goat anti-Rabbit IgG Thermo Fisher A-21244 1818084
Calnexin ENZO ADI-SPA-860
TOM20 (FL-145) SantaCruz sc-11415
IP3R1 (CT1) custom made, Joseph SK, Samanta S. 1993, PMID: 8384211
IP3R2 custom made, Pocono Rabbit Farms and Laboratories
IgG Nanogold Nanoprobes, 2001-0.5ml 06D222
TOM20 Proteintech 11802-1-AP
AF594 anti-rabbit IgG Molecular Probes A-11037
Abberior STAR RED, goat anti-mouse IgG, 500 µl (1 mg/ml) STRED-1001-500UG

Validation

Commercially available antibodies were validated by the manufacturers in different molecular biological applications. References for applications are provided by the manufacturers. We validated the following antibodies for application: α-FLAG antibodies with Immuno-EM, confocal and STED microscopy comparing FLAG tagged protein expressing to non-expressing cells, shown as control data in the manuscript. α-TOM20 was validated with immune-EM, data not shown. α-IP3R1 was validated before (Joseph SK, Samanta S. 1993, PMID: 8384211) and for this study, comparing IP3R1 expressing wild-type, knockout and rescued KO cell lines with confocal microscopy, as well as with western blot, both shown in the manuscript. α-IP3R2 was validated with western blot, comparing IP3R2 expressing wild-type, knockout and rescued KO cell lines, shown in the manuscript.

Eukaryotic cell lines

Policy information about cell lines

Cell line source(s)

DT40 wild-type and IP3 deficient TKO cells were provided by Prof. Tomohiro Kurosaki (RIKEN Research Center for Allergy and

Cell line source(s)	Immunology, Yokohama, Japan). IP3R expressing stable clones from TKO DT40 cells were produced by David I. Yule, (University of Rochester, Rochester, NY). WT and IP3 deficient TKO HeLa cells were provided by Katsuhiko Mikoshiba (RIKEN Brain Science Institute, Saitama, Japan).
Authentication	none of the cell lines were authenticated
Mycoplasma contamination	All cell lines were tested negative for micoplasma
Commonly misidentified lines (See ICLAC register)	No commonly misidentified lines were used in this study.
